# Biobased Random Copolymers From Furandicarboxylic Acid Isomers: Proof of Concept for an Integrated Biorefinery Approach Toward Outstanding Sustainable Food Packaging

**DOI:** 10.1002/gch2.202600003

**Published:** 2026-04-24

**Authors:** Enrico Bianchi, Giulia Guidotti, Michelina Soccio, Valentina Siracusa, Shanmugam Thiyagarajan, Nadia Lotti

**Affiliations:** ^1^ Department of Civil Chemical Environmental and Materials Engineering University of Bologna Bologna Italy; ^2^ Interdepartmental Center for Industrial Research on Advanced Applications in Mechanical Engineering and Materials Technology CIRI‐MAM Bologna Italy; ^3^ Interdepartmental Center for Industrial Research on Buildings and Construction CIRI‐EC Bologna Italy; ^4^ Department of Chemical Science University of Catania Catania Italy; ^5^ Wageningen Food & Biobased Research Wageningen University & Research Wageningen The Netherlands; ^6^ Interdepartmental Center for Industrial Agro‐Food Research CIRI‐AGRO Cesena Italy

**Keywords:** 2,4‐FDCA, 2,5‐FDCA, copolymerization, polyester, renewable resources

## Abstract

This work explored the industrial potential of an alternative green synthetic route to obtain 2,5‐furandicarboxylic acid (2,5‐FDCA) via a Henkel‐type disproportionation reaction developed by Thiyagarajan et al., meant to produce furan, and up to 30% of 2,4‐furandicarboxylic acid (2,4‐FDCA), a structural isomer of 2,5‐FDCA. Linear glycols were combined with FDCA isomers from the Henkel‐type reaction to synthesize three fully biobased random copolymers: 2,5‐2,4‐PTF, 2,5‐2,4‐PBF, 2,5‐2,4‐PHF. These copolymers were compression molded and subjected to NMR, viscometry, WAXS, DSC, and TGA analyses. Evidence suggested the formation of a partially ordered phase in 2,5‐2,4‐PHF. Mechanical and gas barrier properties of the synthesized copolymers were remarkably superior to the ones of both the reference homopolymers, with increased toughness, elongation at break and resistance to humidity. Interestingly, humidity improved the gas barrier performance of 2,5‐2,4‐PBF, making it impermeable to CO_2_. These findings highlighted the potential of these furan‐based copolyesters for the production of mono‐material, potentially recyclable, and sustainable food packaging. These achievements represented promising proof of concept for an integrated biorefinery and polymerization process, designed to: 1) Completely avoid the use of solvents; 2) Start from second‐generation biomass; 3) Have high carbon efficiency and few purification steps.

## Graphical abstract

An integrated, solvent‐free route paired a Henkel‐type synthesis of biobased FDCA isomers with their copolymerization using linear glycols, to yield three random copolyesters (2,5‐2,4‐PTF, 2,5‐2,4‐PBF, 2,5‐2,4‐PHF). Compression‐molded films showed higher toughness, elongation, and humidity‐resistant gas barriers (impermeability to CO_2_ for PBF), enabling recyclable, mono‐material packaging. This proof‐of‐concept suggested potential for green biorefinery coupled with polymerization.

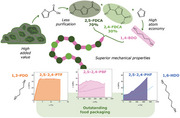

## 1 Introduction

**SCHEME 1:**
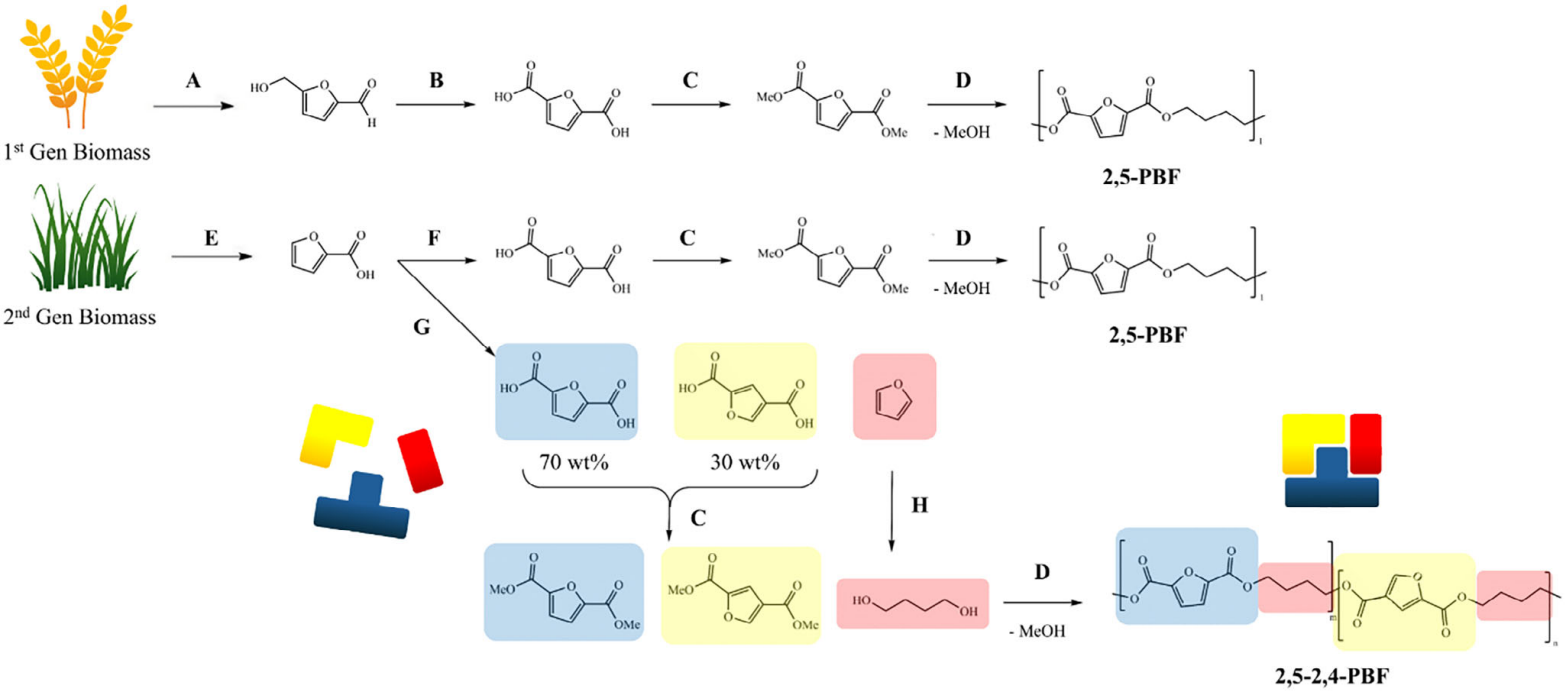
The process under study, compared to two other state‐of‐the‐Art examples of production of furan‐based polyesters [40]. A: extraction, hydrolysis, dehydration; B: oxidation; C: esterification; D: polymerization; E: extraction, hydrolysis, dehydration, oxidation; F: carboxylation; G: Henkel‐type disproportionation; H: hydrogenation, hydrogenolysis.

Plastics are of paramount importance to our society due to their versatility, lightweight nature, and cost‐effectiveness. However, the high carbon footprint, the limited degradability, and thus, the long permanence in the environment of many fossil‐based plastics have been raising great ecological concerns, taking also into account the acceleration of plastic production, which is projected to grow worldwide from about 414 million tons in 2023 to 1800 million tons in 2050 [1, 2]. Therefore, the development of polymers from renewable resources, otherwise called biobased polymers, has become an appealing alternative and a fundamental starting point in the eco‐design of new sustainable materials. In fact, shifting from petrochemical feedstocks to biomass has the potential to mitigate the environmental issues and carbon emissions associated with the extraction and the inevitable depletion of fossil resources [3, 4, 5]. Unlike petrochemical refineries, biorefineries are independent from the fluctuations of the petrochemical markets and represent a more sustainable approach toward the production of chemicals [6, 7]. In particular, life cycle assessments of many biorefinery scenarios were carried out and great potential was found in second‐generation biomass [8], i.e., biomass from plant‐based and food waste. The valorization of waste into high‐added‐value chemicals is the goal of research in many fields, including polymer chemistry. One of the most promising biobased chemicals under study in recent years is 2,5‐furandicarboxylic acid (2,5‐FDCA), because of its potential to be used as a building block for the production of several other chemicals and polymers [9, 10]. Specifically, furan‐based biopolymers have emerged in the field of food packaging due to their exceptional functional properties [11, 12, 13, 14, 15, 16, 17, 18]: mono‐material furan‐based films were found to have excellent thermal stability and mechanical performances. In addition, their gas barrier properties are in some cases comparable to the ones of ethylene vinyl alcohol) (EVOH), the industrial standard in terms of gas barrier properties in multilayered food packaging films [19]. These properties have been correlated with the formation of a partially ordered 1D/2D mesomorphic phase, which was experimentally observed, particularly in the case of poly(pentamethylene 2,5‐furanoate) (2,5‐PPeF) [20, 21, 22]. Consequently, these biopolymers were found to reach unparalleled levels of performance in this field, making them very appealing candidates for the replacement of their traditional petrochemical counterparts, such as poly(ethylene terephthalate) (PET) [23, 24, 25, 26, 27, 28, 29, 30, 31, 32]. Although the catalytic dehydration of C_6_ sugars to 5‐hydroxymethylfurfural (HMF), followed by aerobic oxidation of 5‐hydroxymethylfurfural (HMF) remains the most extensively investigated pathway for 2,5‐FDCA production [14, 33], alternative routes have recently gained traction to diversify feedstock utilization and avoid oxidation. Routes starting from galactose‐rich biomass allow the production of galactaric acid, which can be subjected to double dehydration and cyclization, offering advantages in terms of selectivity and reduced formation of unstable aldehyde intermediates [34, 35, 36]. Another emerging approach involves the carboxylation of furoic acid through CO_2_ addition, either via Kolbe–Schmitt‐type reactions or metal‐catalyzed C–H carboxylation, providing an attractive pathway that directly incorporates CO_2_ as a carbon source [37, 38, 39]. An additional sustainable synthetic route to obtain 2,5‐FDCA from second‐generation biomass was studied, based on a Henkel‐type disproportionation reaction [40]. The concurrent formation of up to 30% of 2,4‐furandicarboxylic acid (2,4‐FDCA), the structural isomer of 2,5‐FDCA, was observed. As a consequence, 2,4‐FDCA attracted the attention of the scientific community, leading to the synthesis and characterization of homopolymers and copolymers based on 2,4‐FDCA [41, 42, 43, 44, 45, 46, 47]: all poly(alkylene 2,4‐furanoate)s (2,4‐PAFs) were superior than poly(alkylene 2,5‐furanoate)s (2,5‐PAFs) in terms of mechanical properties, and their gas barrier performances were found to be comparable and sometimes far superior than the ones of 2,5‐PAFs [47]. These results were attributed to the possible formation of a more compact mesomorphic phase due to the different geometry of 2,4‐FDCA, which might cause the furan oxygen to be more accessible for the establishment of interchain polar interactions, responsible of the formation of a partially ordered microstructure [48]. Within the context of this research, it was observed how the Henkel‐type reaction had great potential for the design of a process which could integrate the biorefinery of FDCA with the production of high‐added‐value polyesters. In fact, furan, which is a side product of the Henkel‐type reaction, is already industrially used for the production of 1,4‐butanediol [49]. Thus, in the present work, a random copolymer from 2,5‐FDCA, 2,4‐FDCA, and 1,4‐butanediol was studied. In order to assess the effect of the glycolic chain length on the properties of this emerging class of copolymers, 1,3‐propanediol and 1,6‐hexanediol were also used. A schematic representation of this work and the reaction products is shown in Scheme 1. Three copolymers were obtained as the final product: poly(trimethylene 2,5‐furanoate/2,4‐furanoate) (2,5‐2,4‐PTF), poly(butylene 2,5‐furanoate/2,4‐furanoate) (2,5‐2,4‐PBF), and poly(hexamethylene 2,5‐furanoate/2,4‐furanoate) (2,5‐2,4‐PHF). These materials were compression molded into films and subjected to a complete characterization of their molecular, structural, thermal, mechanical, and gas barrier properties .

## 2 Experimental

### 2.1 Materials

Dimethyl 2,4‐furandicarboxylate and dimethyl 2,5‐furandicarboxylate (2,4‐DMF and 2,5‐DMF, respectively) were synthesized following the method reported in the literature [40]. Additionally, the following materials were used as purchased: 1,3‐propanediol (1,3‐PD) (97% purity; Carbosynth Ltd, Combrook, UK); 1,4‐butanediol (1,4‐BD) (97% purity; Sigma–Aldrich, Saint Louis, MO, USA); 1,6‐hexanediol (1,6‐HD) (97% purity; Tokyo Chemical Industry Co. Ltd, Tokyo, Japan); titanium (IV) butoxide (TBT) (Sigma–Aldrich, Saint Louis, MO); titanium (IV) isopropoxide (TIP) (Sigma–Aldrich, Saint Louis, MO); deuterated chloroform containing 0.03 v/v% tetramethylsilane as internal standard (99.8% purity; Eurisotop, Saint‐Aubin, France); trifluoroacetic acid (99.5% purity; J.T. Baker, Phillipsburg, NJ, USA); phenol/1,1,2,2‐tetrachloroethane 60:40 wt.% mixture (Sigma–Aldrich, Saint Louis, MO).

### 2.2 Synthesis and Film Preparation

For the synthesis of 2,5‐2,4‐PTF, the following reagents were used: 1.41 g (7.7 mmol) of 2,4‐DMF, 3.29 g (17.9 mmol) of 2,5‐DMF, 3.88 g (51.0 mmol, 99 mol% excess compared to the diacids) of 1,3‐PD, 200 ppm of TIP, and 200 ppm of TBT. For the synthesis of 2,5‐2,4‐PBF, the following reagents were used: 1.32 g (7.1 mmol) of 2,4‐DMF, 3.07 g (16.7 mmol) of 2,5‐DMF, 3.65 g (40.5 mmol, 70 mol% excess compared to the diacids) of 1,4‐BD, 200 ppm of TIP, and 200 ppm of TBT. For the synthesis of 2,5‐2,4‐PHF, the following reagents were used: 0.83 g (4.5 mmol) of 2,4‐DMF, 1.94 g (10.5 mmol) of 2,5‐DMF, 3.02 g (25.5 mmol, 70 mol% excess compared to the diacids) of 1,6‐HD, 200 ppm of TIP, and 200 ppm of TBT. For each synthesis, the reagents and catalysts were charged inside a 250 mL glass reactor, which was closed with a three‐neck head and placed inside a silicone oil bath. During the first stage, the temperature of the bath was set at 180°C and the system was kept under mechanical stirring at a speed of 100 rpm, under constant nitrogen flow. The esters melted and dissolved into the glycols readily (less than 15 min). Once the content of the reactor looked like a transparent solution, the system was attached to a trap apparatus to collect methanol, the byproduct of the polycondensation reaction. This stage lasted approximately 2 h, until 90% of the theoretical methanol was collected. During the second stage, the temperature of the bath was gradually raised to 220°C while the pressure inside the system was gradually lowered to 0.05 mbar, over the course of an hour. The molecular weight of the polymer increased by removing the glycolic excess. As soon as the system reached the maximum temperature and minimum pressure, it was kept under stirring for about 1.5 h, while monitoring the torque. Once the torque reached a plateau, the reaction was considered concluded.

The so‐obtained polymeric samples were compression molded using a C12 laboratory press (Carver, Wabash, IN). 2.5 g of each sample was placed between two Teflon sheets and positioned inside the press, set at a temperature 30°C higher than the melting temperature (for semicrystalline samples) or the glass‐to‐rubber transition (for amorphous samples). After 1 min, a pressure of 9 ton/m^2^ was applied gradually, over the course of 1 min. Finally, the pressure was released, and the sample was cooled under a metal plate at room temperature, to obtain a film with a diameter of 11 cm and thickness around 300 µm. Film samples were stored for at least two weeks in a desiccator at room temperature, before their characterization.

### 2.3 Molecular Characterization

Nuclear Magnetic Resonance Spectroscopy (NMR) experiments were conducted with a 400‐MHz Varian Inova (Agilent Technologies, Palo Alto, CA, USA). For ^1^H‐NMR experiments, 10 mg of sample were solved in 0.7 mL of deuterated chloroform containing few drops of trifluoroacetic acid. For ^13^C‐NMR experiments, 40 mg of sample were used for the analyses.

Intrinsic viscosity (η) experiments were conducted with a 31 13/Ic Ubbelohde viscometer apparatus with a diameter of 0.84 mm, inserted in a thermostated water bath set at 30°C, and connected to an electronic sensor for the flow‐time measurements. Solutions of polymer in a phenol:1,1,2,2‐tetrachloroethane 60:40 wt.% mixture were prepared at four different concentrations (0.80, 0.69, 0.58, and 0.47 g/dL) and five measurements were carried out on each solution. For each measurement, the relative viscosity (ɳ_r_) and specific viscosity (ɳ_sp_) were calculated, and their respective linear plots vs concentration were obtained. ɳ was extrapolated from these plots.

### 2.4 Thermal Characterization

Differential Scanning Calorimetry (DSC) experiments were conducted with a DSC6 (PerkinElmer, Waltham, MA). Approximately 5 mg of sample were placed in an aluminum pan, then subjected to a heating‐cooling‐heating cycle under a nitrogen flow (20 mL/min). The heating scans were programmed from −20 to 150°C at 20°C/min, while the cooling scan was programmed from 150 to −20°C at 100°C/min. The glass transition temperature (T_g_) was calculated as the midpoint between the baselines of the glass transition inflection, while the specific heat increment (Δc_p_) was calculated as the difference between the two baselines. The melting temperature (T_m_) was calculated as the peak maximum, while the melting enthalpy (ΔH_m_) as the integral of the peak, respectively.

Thermogravimetric Analysis (TGA) experiments were conducted with a TGA4000 (PerkinElmer, Waltham, MA). Approximately 10 mg of sample were placed in an alumina pan, then subjected to a heating scan from 40 to 800°C at 10°C/min, under pure nitrogen flow (40 mL/min). The degradation onset temperature (T_onset_) was calculated as the weight loss onset. The maximum degradation temperature (T_max_) was calculated as the minimum of the derivative of the thermogram.

### 2.5 Structural Characterization

Wide Angle X‐ray Scattering (WAXS) experiments were conducted with an X'Pert diffractometer (PANalytical, Almelo, The Netherlands), equipped with a PIXcel detector and a copper source with wavelength of 0.154 nm. The detector moved in 0.1° steps, at a rate of 100 s/step. The crystalline area (A_c_) was calculated by considering the total area (A_t_) and subtracting the amorphous halo from it. The degree of crystallinity (X_c_) was calculated as the ratio A_c_/A_t_. Incoherent scattering was not taken in consideration. WAXS experiments were also carried out during a temperature scan, at a rate of 20°C/min, with a TTK450 heating device (Anton Paar, Gratz, Austria).

### 2.6 Mechanical Tests

Tensile tests were conducted with an Instron 5966 (Instron, Norwood, MA) equipped with a transducer‐coupled 10 kN load cell. Film samples were cut in strips of 0.5 cm width and gauge length of 2 cm. The thickness of each sample was measured before testing. The samples were stretched at a rate of 10 cm/min. The results were converted into stress‐strain plots. The elastic modulus (E) was calculated as the slope of the linear elastic region. The yield elongation and stress (ε_y_ and σ_y_, respectively) were determined as the coordinates of the yield point. The elongation and stress at break (ε_b_ and σ_b_, respectively) were determined as the coordinates of the breaking point. Toughness was estimated as the integral of the area underneath the stress‐strain curve. For each material under study, 5 or more samples were tested, and the mechanical characterization data are reported as mean value ± standard deviation. The stress‐strain curves shown in the Figures of this work were chosen as the most representative of the mean values.

### 2.7 Gas Barrier Tests

Gas barrier tests were carried out with a GDP‐C permeance testing device (Brugger Feinmechanik, Munchen, Germany) equipped with an HAAKE DC10‐K15 external thermostat (ThermoFisher Scientific, Waltham, MA, USA). Film samples with diameter of 10 cm were tested against pure O_2_ or CO_2_, at a temperature of 23°C, two different values of relative humidity (of 0% and 85%, respectively), gas flow of 100 cm^3^/min, gas pressure of 1 atm. The procedure followed the manometric method found in *Gas Permeability Testing Manual* and in the standards *ASTM D1434‐82(2009)*, *DIN 53536*, and *ISO/DIS 15105‐1:2007*. The results were converted into pressure‐time plots. From them, the Gas Transmission Rate (GTR) values were calculated. All gas transmission values reported in this work, both measured experimentally and retrieved from the literature, were obtained in the same standardized conditions. For this reason, they are comparable.

## 3 Results and Discussion

### 3.1 Molecular Characterization

**FIGURE 1:**
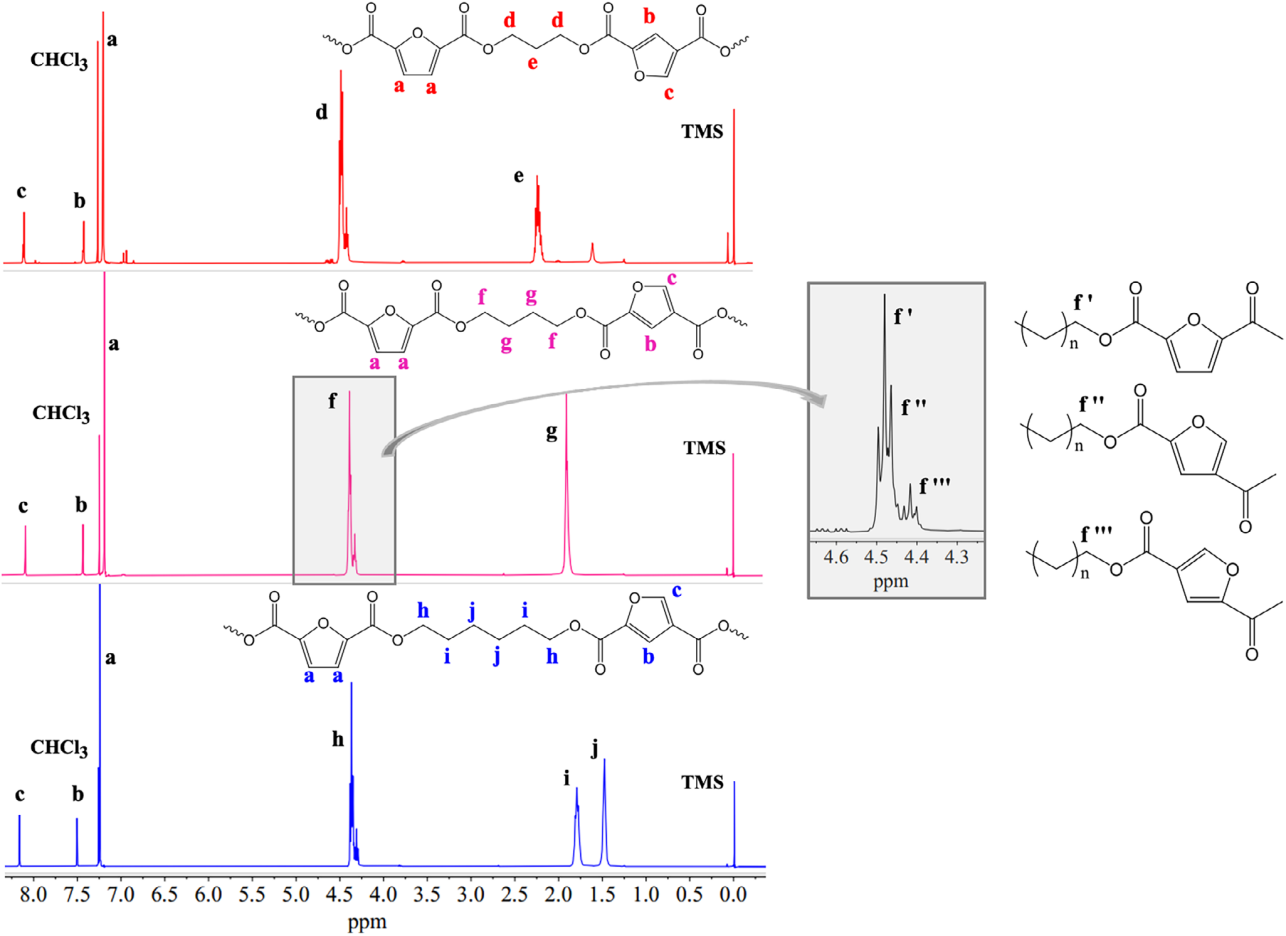
From top to bottom, ^1^H‐NMR spectra of 2,5‐2,4‐PTF, 2,5‐2,4‐PBF, and 2,5‐2,4‐PHF.

After synthesis, 2,5‐2,4‐PAF copolymers (Figures S1, S2, S3), were subjected to ^1^H‐NMR spectroscopy analyses to verify their chemical structure and composition. The monomers were also analyzed, as shown in Figure S4. The results are shown in Table 1 and Figure 1, together with the attribution of the peaks. For all the experiments, the aromatic (furanic) signals of the copolymers had a similar chemical shift [19, 47]. In the case of ^1^H‐NMR, it must be specified that in all spectra, the peak at 7.26 ppm corresponded to non‐deuterated chloroform traces. The signal originated by trifluoroacetic acid was located at about 11.50 ppm. In the glycol subunits, triplets ascribed to the –OCH_2_– moieties in the region 4.5–4.25 ppm (labeled as *d* for 2,5‐2,4‐PTF*, f* for 2,5‐2,4‐PBF and *h* for 2,5‐2,4‐PHF in Figure 1) and also multiples associated to the inner methylene groups in the region 2.25–1.5 ppm (labeled as *e* for 2,5‐2,4‐PTF*, g* for 2,5‐2,4‐PBF, *i* and *j* for 2,5‐2,4‐PHF in Figure 1) can be detected. The triplets coming from ‐OCH_2_‐ split into three signals depending on the nature of FDCA moiety, i.e., 2,5‐FDCA or 2,4‐FDCA isomers, and on the orientation of the 2,4‐FDCA subunit. The latter is not symmetric, unlike the 2,5‐FDCA subunit. In the insert of Figure 1 it is shown, as an example, a magnification at 4.7–4.2 ppm of the spectrum of 2,5‐2,4‐PBF. In the aromatic ring, all 2,5‐2,4‐PAF copolymers showed a singlet (*c*) at 8.00–8.16 ppm, which corresponds to the furan hydrogen atom in the 5‐position of the furan ring in 2,4‐FDCA subunits, while the singlet (*b*) at 7.42–7.50 ppm corresponds to the aromatic hydrogen atom in the 3‐position of the furan ring in 2,4‐FDCA subunits. The singlet (*a*) at 7.15–7.24 ppm is originated by the hydrogen atoms belonging to the 3‐ and 4‐positions of the furan ring, in the 2,5‐FDCA subunit. The composition of 2,5‐2,4‐PAF copolymers was verified considering the normalized area under the *a* and *b* singlets, relative to 2,5‐FDCA and 2,4‐FDCA isomers, respectively. The results shown in Table 1 confirmed the achievement of a composition remarkably close to the reaction feed, confirming that the reactivity of two isomers of FDCA are very similar.

**TABLE 1:** Molecular characterization data: Feed composition, found ^1^H‐NMR composition, and intrinsic viscosity (ɳ).

	Feed	Found	Viscometry
	2,4‐isomer (mol%)	2,4‐isomer (mol%)	ɳ (dL/g)
**2,5‐2,4‐PTF**	30.1	28.4	0.87
**2,5‐2,4‐PBF**	29.8	29.1	1.10
**2,5‐2,4‐PHF**	30.0	29.3	1.06

**FIGURE 2:**
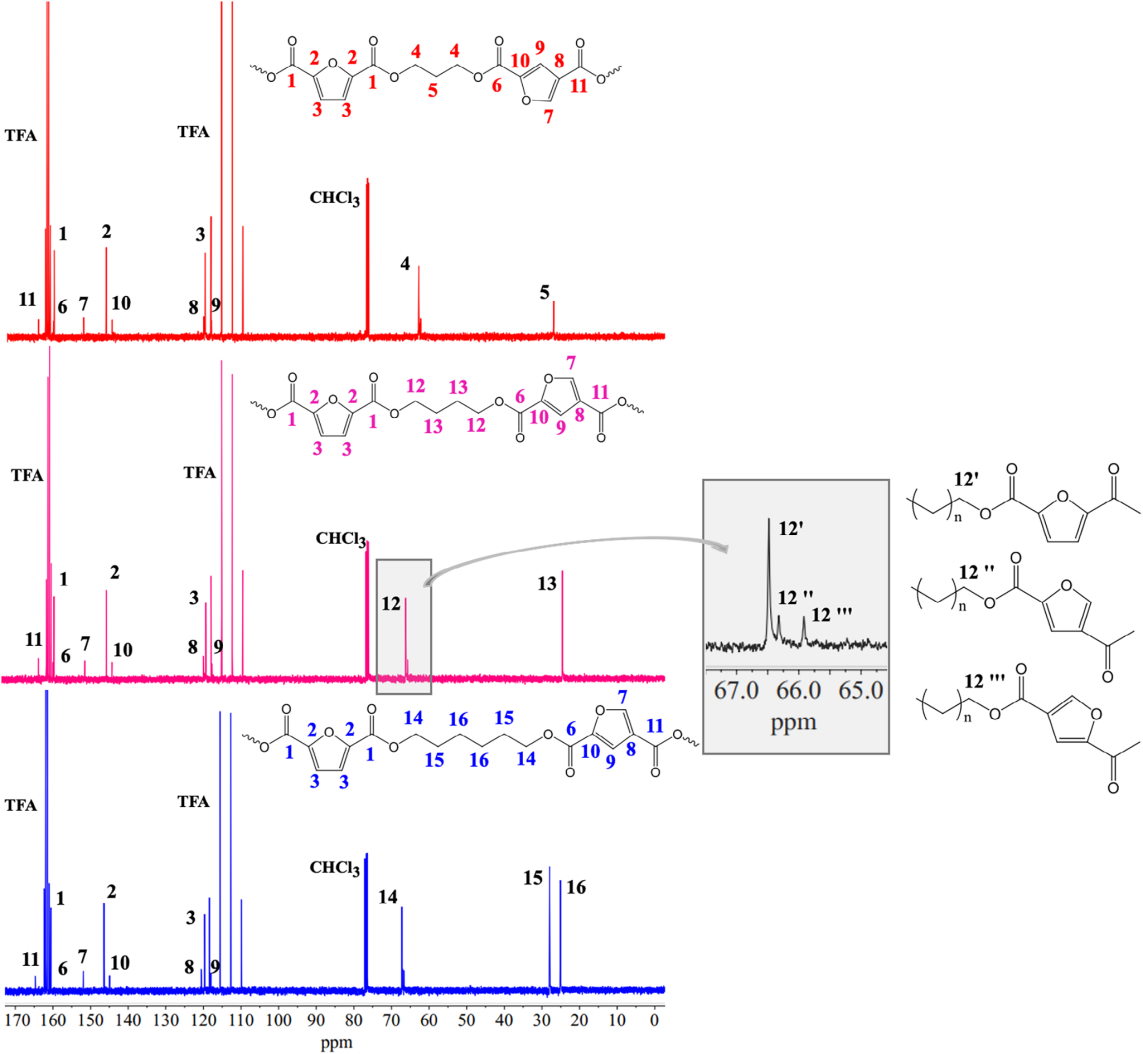
From top to bottom, ^13^C‐NMR spectra of 2,5‐2,4‐PTF, 2,5‐2,4‐PBF, and 2,5‐2,4‐PHF.

^13^C‐NMR experiments were also carried out: the corresponding spectra are shown in Figure 2, together with the attribution of the peaks. The signals arising from the solvents (CDCl_3_ and TFA) are also labeled. The peak coming from the –OCH_2_– carbons splits into three signals depending on the nature of the FDCA moiety, i.e., 2,5‐FDCA or 2,4‐FDCA isomers, and depending on the orientation of the asymmetric 2,4‐FDCA group. In the insert of Figure 2, it is shown, as an example, a magnification at 67–65 ppm of the spectrum of 2,5‐2,4‐PBF.

Overall, NMR experiments confirmed that the synthetic protocol allowed the achievement of good control over the polymeric architecture of the copolymers under study.

An indication about the molecular weight of 2,5‐2,4‐PAF samples was obtained with viscometric measurements. The results, expressed as intrinsic viscosity (η), are summarized in Table 1. The η values are all high and close to 1, indicating that this parameter could be ignored when comparing the solid‐state and functional properties of the copolymers under study. This observation was corroborated by the measurement of the integrals of the ^1^H‐NMR spectra: in fact, signals d, f, and h (of 2,5‐2,4‐PTF, 2,5‐2,4‐PBF, and 2,5‐2,4‐PHF, respectively) from Figure 1 had in their proximity much smaller triplets which can be attributed to the hydrogen atoms of the glycolic methylene groups at the terminals of the polymer chains. The total integral of these smaller triplets was a small fraction of the integral of signals d, f, and h. Specifically, terminals were 8%, 5%, and 3% of the main signal for 2,5‐2,4‐PTF, 2,5‐2,4‐PBF, and 2,5‐2,4‐PHF. This result confirms the fact that all polymers under study had high molecular weight and that 2,5‐2,4‐PBF and 2,5‐2,4‐PHF had slightly higher molecular weight compared to 2,5‐2,4‐PTF.

### 3.2 Thermal Characterization

**FIGURE 3:**
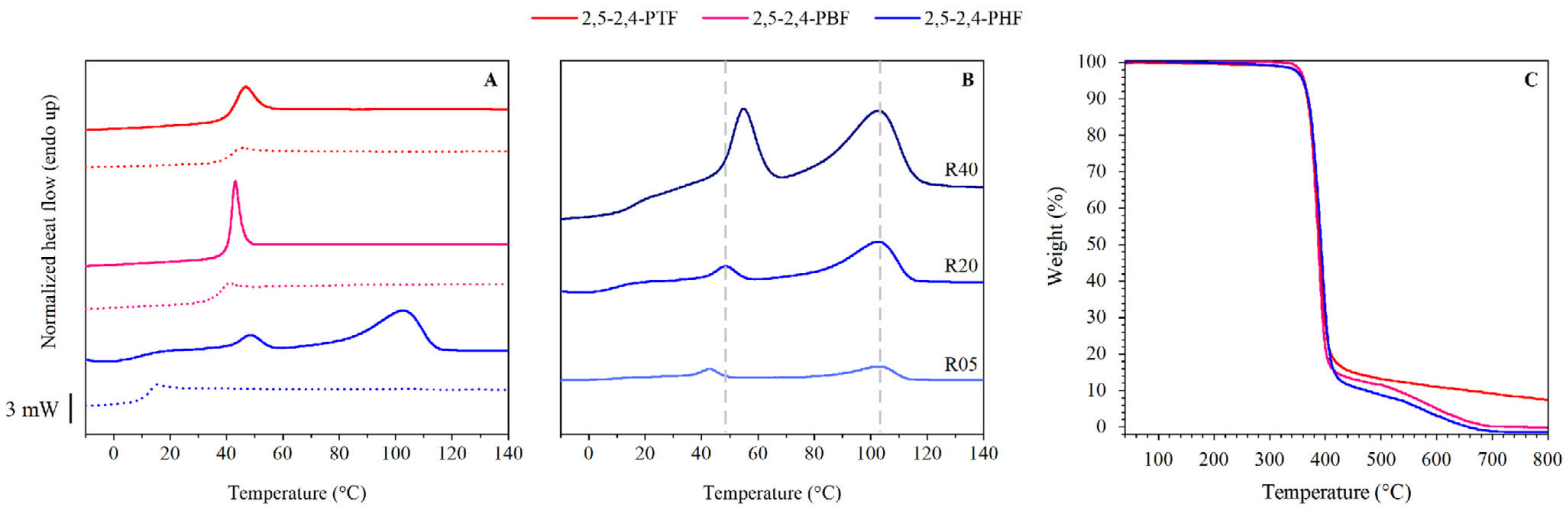
Panel A: first (solid line) and second (dotted line) DSC scan at 20°C/min on 2,5‐2,4‐PTF, 2,5‐2,4‐PBF, and 2,5‐2,4‐PHF. Panel B: first DSC scan at varying heating rate on 2,5‐2,4‐PHF. Panel C: TGA curves of 2,5‐2,4‐PTF, 2,5‐2,4‐PBF, and 2,5‐2,4‐PHF.

2,5‐2,4‐PAF copolymers were thermally characterized through Differential Scanning Calorimetry (DSC) and Thermogravimetric Analysis (TGA). Data from the first and second heating scans are reported in Table 2, and their corresponding curves are shown in Figure 3, panel A. Both 2,5‐2,4‐PTF and 2,5‐2,4‐PBF exhibit the typical behavior of amorphous materials, displaying only the endothermic change in baseline related to the glass‐rubber transition. Furthermore, both copolymers are glassy at room temperature, and immediately after T_g_ they show the phenomenon of physical aging. 2,5‐2,4‐PHF was the only semicrystalline copolymer within the family, displaying a glass transition below room temperature, corresponding to a rubbery amorphous phase, followed by two endothermic peaks at 48°C and 102°C. The exact nature of these two peaks was investigated by carrying out DSC experiments at different heating rates, of 5, 20, and 40°C/min, respectively. The corresponding thermal data is shown in Table 2, while the DSC traces are represented in Figure 3, panel B. As can be noted, the higher‐melting peak did not change its position, while the other peak shifted its position toward higher temperatures by increasing the heating rate. It is known that, while a first‐order transition such as the melting of crystals does not change its position if the heating rate is changed, a shift in position is typical of second‐order phenomena, such as a glass‐to‐rubber transition or the isotropization of a mesophase, i.e., a phase with a short‐range order. Specifically, the presence in furan‐based polyesters of a 1D/2D, partially ordered mesomorphic phase has already been investigated in the literature via WAXS and SAXS experiments [20], and it is reasonable to believe that the second order transition in 2,5‐2,4‐PHF might have been originated by a similar structure. Interestingly, the position of this second‐order peak is similar to the one found in the 2,4‐PHF homopolymer, for which evidence was also gathered, to support the claim of a mesomorphic structure [47]. The position of the peak in the homopolymer and in the copolymer was 58°C and 48°C, respectively. Moreover, the positions of the T_g_ and of the isotropization temperature might suggest a smectic structure, as nematic ones are typically found above T_m_ in semicrystalline polyesters [50, 51, 52, 53]. The melting temperature of 2,5‐2,4‐PHF was 102°C, well below the one of 2,5‐PHF homopolymer (144°C): the crystals in 2,5‐2,4‐PHF should be primarily constituted by 2,5‐PHF segments, but with a lower degree of perfection caused by the presence of 2,4‐PHF ones. Because of this, the melting enthalpy of 2,5‐2,4‐PHF was found to be lower than the one of 2,5‐PHF [19, 47], indicating a limited ability to crystallize due to 2,4‐PHF counits, which are presumably rejected out of the crystal lattice. This was particularly expected since, unlike 2,5‐FDCA subunits, 2,4‐FDCA subunits have no plane of symmetry and can polymerize in two different orientations (Figure 1), increasing the irregularity of the corresponding repeating units and making the crystallization of 2,5‐2,4‐PHF less favorable.

**TABLE 2:** Thermal (DSC, TGA) and diffractometric (WAXS) characterization data on 2,5‐2,4‐PTF, 2,5‐2,4‐PBF, 2,5‐2,4‐PHF, and their respective parent homopolymers [19, 47].

			DSC				WAXS	TGA	
			T_g_ (°C) Δc_p_ (J/g°C)	T_cc_ (°C) ΔH_cc_ (J/g)	T_m1_ (°C) ΔH_m1_ (J/g)	T_m2_ (°C) ΔH_m2_ (J/g)	X_c_ (%)	T_onset_ (°C)	T_max_ (°C)
**PTF**									
**2,5‐PTF** [19]	**I scan**		52 0.361	136 7	169 7	—	0	364	386
	**II scan**		52 0.359	—	—	—			
**2,4‐PTF** [47]	**I scan**		38 0.394	—	—	—	0	390	409
	**II scan**		40 0.511	—	—	—			
**2,5‐2,4‐PTF**	**I scan**		43 0.382	—	—	—	0	370	390
	**II scan**		42 0.387	—	—	—			
**PBF**									
**2,5‐PBF** [19]	**I scan**		39 0.243	102 26	170 35	—	∼0	382	407
	**II scan**		39 0.281	107 30	170 35	—			
**2,4‐PBF** [47]	**I scan**		33 0.376	—	—	—	0	396	409
	**II scan**		33 0.337	—	—	—			
**2,5‐2,4‐PBF**	**I scan**		38 0.353	—	—	—	0	373	389
	**II scan**		36 0.448	—	—	—			
**PHF**									
**2,5‐PHF** [19]	**I scan**		13 0.205	—	144 40	—	23 ± 2	384	404
	**II scan**		13 0.301	—	144 39	—			
**2,4‐PHF** [47]	**I scan**		9 0.282	—	58 23	—	13 ± 0.1	391	409
	**II scan**		9 0.471	—	—	—			
**2,5‐2,4‐PHF**	**I scan**	**R05**	6 0.216	—	44 2	102 6	12 ± 2	371	392
		**R20**	10 0.237	—	48 2	102 25			
		**R40**	17 0.176	—	55 20	102 44			
	**II scan**		13 0.461	—	—	—			

Following the first scan, a second scan was conducted after rapid cooling of the polymeric melt. The corresponding curves are reported in Figure 3, panel A, as dotted lines. In all cases, the rapid cooling successfully suppressed the ability of 2,5‐2,4‐PAF copolymers to crystallize, and their heating scans only showed their glass‐to‐rubber transitions. To predict the T_g_ of random copolymers having a different 2,5‐/2,4‐isomer composition, it was possible to fit the T_g_ values obtained during the second DSC heating scan on the Gordon–Taylor equation [54]:

(1) $$T_{g}=\frac{W_{A}\cdot T_{gA}+k\cdot W_{B}\cdot T_{gB}}{W_{A}+k\cdot W_{B}}\;$$

Specifically, the T_g_ of the reference homopolymers and the T_g_ of the corresponding copolymer were fitted on the Gordon–Taylor equation, adjusting the k parameter to make the coefficient of determination (R^2^) equal to 1. The best fit was obtained by setting the k parameter to 12.57, 2.00, and 0.01 for 2,5‐2,4‐PTF, 2,5‐2,4‐PBF, and 2,5‐2,4‐PHF, respectively. More random copolymers with different 2,5‐/2,4‐isomer compositions will be needed to confirm the validity of this predictive model.

TGA analyses were also conducted under inert atmosphere to identify the degradation temperatures of 2,5‐2,4‐PAF copolymers. The T_onset_ and T_max_ values are collected in Table 2, while the corresponding thermograms are shown in Figure 3, panel C. In all cases, thermal stability was high and comparable for all the polymers under study, with T_onset_ ranging between 370°C and 372°C, and T_max_ in the range of 389°C–392°C. The thermogram for 2,5‐2,4‐PTF exhibited a single‐step degradation profile, while the other two curves showed a two‐step profile, the first just above 370°C, and the second at higher temperatures (around 500°C), suggesting a different degradation mechanism. This result may be attributed to the number of carbons in the glycolic subunit, since different degradation profiles were observed in the literature for 2,5‐PAF homopolymers with glycolic subunits having an even or odd number of methylene groups [19]. It is also interesting to compare the residual TGA weight for the copolymers under study and their 2,5‐PAF homopolymers of reference: similarly to 2,5‐PBF and 2,5‐PHF, 2,5‐2,4‐PBF and 2,5‐2,4‐PHF lose almost 100% of their initial weight, and similarly to 2,5‐PTF, 2,5‐2,4‐PTF retains about 10% of its initial weight. Overall, the copolymers were found to have slightly lower thermal stability than the homopolymers of reference, with T_onset_ and T_max_ values 10°C–20°C lower, but still sufficiently high to prevent degradation phenomena during processing.

### 3.3 Structural Characterization

**FIGURE 4:**
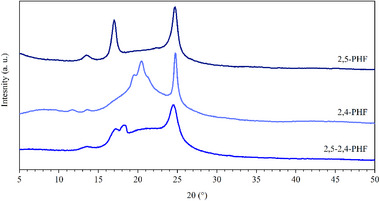
WAXS diffraction profile on 2,5‐2,4‐PHF and its parent homopolymers [19, 47].

In order to study the crystalline structure of the compression‐molded samples under investigation and determine their degree of crystallinity, Wide Angle X‐ray Scattering (WAXS) analyses were conducted. Concerning 2,5‐2,4‐PTF and 2,5‐2,4‐PBF, no reflections indicative of a crystalline phase was observed, in accordance with the calorimetric data. In the case of 2,5‐2,4‐PHF, several peaks were found to emerge from the amorphous halo. According to the corresponding diffractogram shown in Figure 4, four reflections were found at 13.6°, 17.2°, 18.3°, and 24.5°. The homopolymers of reference [19, 47] were also compared to 2,5‐2,4‐PHF (Figure 4): in detail, the reflection at 17.2° can be ascribed to the 2,5‐PHF homopolymer, while those at 13.6° and 24.5° are present in both the ordered phases of 2,5‐PHF and 2,4‐PHF. One further reflection, located at 18.3°, was found in 2,5‐2,4‐PHF, but not for the two reference homopolymers. WAXS experiments were also carried out on 2,5‐2,4‐PHF upon heating at 20°C/min, in order to attribute DSC transitions to specific WAXS reflections. However, the WAXS reflections simultaneously and rapidly disappeared at about 100°C, while no change was observed before and after the isotropization temperature located at 48°C. This finding indicates that the crystalline reflections observed for 2,5‐2,4‐PHF should all be associated with a single crystalline phase. The degree of crystallinity was also calculated for 2,5‐2,4‐PHF and it was found to be equal to 12%, in agreement with calorimetric data. This value is comparable to the one calculated for 2,4‐PHF and almost halved with respect to the one of 2,5‐PHF (Table 2).

### 3.4 Mechanical Tests

**FIGURE 5:**
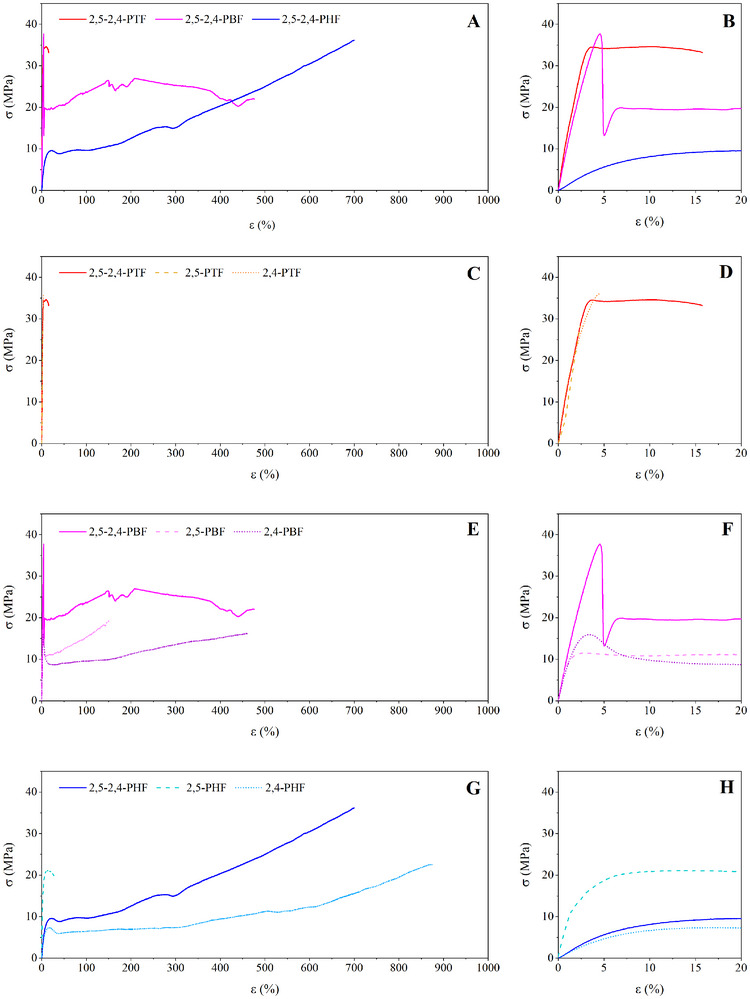
Stress‐strain curves of 2,5‐2,4‐PAF copolymers (panel A) and magnification at low elongation (panel B); stress strain curves of PTF homopolymers compared with 2,5‐2,4‐PTF (panel C) and magnification at low elongation (panel D); stress strain curves of PBF homopolymers compared with 2,5‐2,4‐PBF (panel E) and magnification at low elongation (panel F); stress strain curves of PHF homopolymers compared with 2,5‐2,4‐PHF (panel G) and magnification at low elongation (panel H) [19, 47].

Tensile tests were performed on rectangular film samples of 2,5‐2,4‐PAF copolymers. The mechanical characterization data are reported in Table 3 and Figure 5, panel A and B, along with those of the respective homopolymers of reference. As known, the major factors influencing the mechanical behavior of polymers are molecular weight, chain mobility (T_g_) and degree of crystallinity. Elastic modulus (E) and stress at break (σ_b_) were found to decrease with the increasing length of glycolic subunit, apart from 2,5‐2,4‐PHF, which had the highest σ_b_ value within the family of copolymers, while the opposite trend was found for elongation at break (ε_b_). Specifically, considering the elastic modulus, 2,5‐2,4‐PTF and 2,5‐2,4‐PBF were the most rigid materials, with similar E (1250 and 1200 MPa, respectively), while 2,5‐2,4‐PHF was less rigid, with an elastic modulus about 10 times lower. This behavior could be explained considering that at room temperature the first two materials were glassy, while 2,5‐2,4‐PHF, despite being semicrystalline, was rubbery (Table 2). Conversely, elongation at break was found to be low in 2,5‐2,4‐PTF (about 14%), but significantly higher in 2,5‐2,4‐PBF (almost 500%). Although both materials were glassy and amorphous at room temperature, the second had a lower T_g_ and an intrinsic viscosity value lower than the other two copolymers (Table 2). Finally, all 2,5‐2,4‐PAF copolymers were found to have a yield point at low elongation values (below 10%).

**TABLE 3:** Mechanical characterization data on 2,5‐2,4‐PTF, 2,5‐2,4‐PBF, 2,5‐2,4‐PHF, and their respective parent homopolymers [19, 47].

	E (MPa)	σ_y_ (MPa)	ε_y_ (%)	σ_b_ (MPa)	ε_b_ (%)	Toughness (10^6^ J/m^3^)
**PTF**						
**2,5‐PTF** [19]	1341 ± 123	—	—	29 ± 5	3 ± 1	0.4 ± 0.4
**2,5‐2,4‐PTF**	1248 ± 239	32 ± 5	3 ± 1	30 ± 6	14 ± 3	5 ± 1
**2,4‐PTF** [47]	1429 ± 146	—	—	38 ± 2	4 ± 1	1.2 ± 0.2
**PBF**						
**2,5‐PBF** [19]	1290 ± 140	11 ± 1	3 ± 1	21 ± 3	157 ± 10	21 ± 5
**2,5‐2,4‐PBF**	1202 ± 132	43 ± 5	4 ± 1	28 ± 5	487 ± 115	149 ± 76
**2,4‐PBF** [47]	940 ± 72	16 ± 1	4 ± 1	16 ± 6	564 ± 139	73 ± 19
**PHF**						
**2,5‐PHF** [19]	906 ± 34	—	—	22 ± 1	42 ± 4	5 ± 1
**2,5‐2,4‐PHF**	110 ± 10	24 ± 2	10 ± 1	37 ± 3	701 ± 67	139 ± 14
**2,4‐PHF** [47]	110 ± 22	8 ± 1	19 ± 2	24 ± 6	851 ± 36	114 ± 25

The mechanical behavior of the materials under study was also compared with the one of the respective homopolymers (Table 3 and Figure 5, panel C, D, E, F, G, and H). Both PTF homopolymers showed high elastic modulus and stress at break, paired with low elongation at break (Table 3): in fact, the breaking point was located before the yield point. The lower elongation at break was caused by the limited chain flexibility imparted by the short glycol subunit, containing just 3 methylene groups, which increased the glass transition temperature above room temperature (Table 2). In comparison with its reference homopolymers, 2,5‐2,4‐PTF showed a similar elastic modulus, with a noticeable increase in elongation at break, potentially due to higher molecular weight than its reference homopolymers. In the case of PBF homopolymers, 2,4‐PBF was amorphous due to the lower symmetry of its repeating unit, so it could not form an organized crystalline structure unless annealed [48]. The mechanical behavior of 2,4‐PBF was unusually excellent for an amorphous polymer, showing high elastic modulus and stress at break, accompanied by necking and an elongation at break of over 500%. On the contrary, 2,5‐PBF showed lower elongation at break and its degree of crystallinity justifies its higher elastic modulus. 2,5‐2,4‐PBF was found to show mechanical properties comparable to those of 2,5‐PBF. Finally, in the case of PHF homopolymers, 2,5‐PHF was characterized by elastic modulus about nine times higher than the one of 2,4‐PHF due to its higher degree of crystallinity, with an elongation at break about 20 times lower. The presence of only 30 mol% of 2,4‐PHF units was enough to make the mechanical behavior of 2,5‐2,4‐PHF very similar to the one of 2,4‐PHF. Regardless of their isomeric composition, for both PBF and PHF polymers, even a very small amount of crystalline phase caused impressive decreases in elongation at break, in contrast to what is typically observed for semicrystalline polymers with a low to moderate degree of crystallinity. This finding could be given an interpretation similar to the one discussed in the literature for 2,4‐PBF [48]: the potential formation of a partially ordered 2D mesomorph phase and pi‐pi interactions seem to be favored by the absence of crystallinity, thus lower degrees of crystallinity corresponded to higher elongations at break. This interpretation would also contribute to explaining the massive increase in toughness caused by copolymerization of the two isomeric units (Table 3), apart from 2,5‐2,4‐PTF, for which increased toughness might have been caused by higher molecular weight. Specifically, for 2,5‐2,4‐PTF, toughness was improved by 977% over 2,5‐PTF and by 306% over 2,4‐PTF. For 2,5‐2,4‐PBF toughness increased by 602% over 2,5‐PBF and by 104% over 2,4‐PBF. Finally, for 2,5‐2,4‐PHF, toughness increased by an outstanding 2526% over 2,5‐PHF and by 22% over 2,4‐PHF. Overall, the substitution of 2,5‐FDCA with a small percentage of 2,4‐FDCA was found to cause exceptional improvements in mechanical properties, particularly in comparison with 2,5‐PAF homopolymers.

### 3.5 Gas Barrier Tests

**FIGURE 6:**
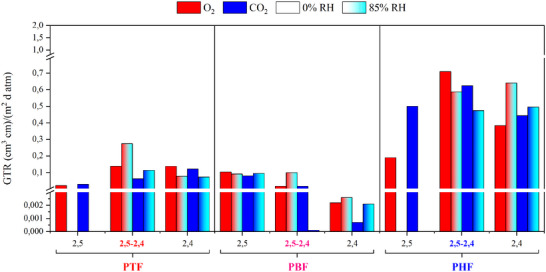
Gas transmission rate values for 2,5‐2,4‐PTF, 2,5‐2,4‐PBF, and 2,5‐2,4‐PHF and their respective parent homopolymers [19, 47]. Data missing for 2,5‐PTF and 2,5‐PHF in humid conditions.

The assessment of gas barrier properties is necessary to understand the potential applications of polymeric materials in the field of packaging, particularly in food packaging, where high performance is required. For this reason, permeability tests on 2,5‐2,4‐PAF copolymers were conducted at 23°C on films obtained via compression molding. Permeability values, expressed as Gas Transmission Rates (GTR), are presented in Table 4 and Figure 6, together with those of the reference homopolymers. Gas barrier properties are influenced by the microstructure of the polymeric film, and specifically, three factors are the most impactful: 1) glass transition temperature, correlated with free volume, since higher free volume can facilitate the passage of gas; 2) degree of crystallinity, since crystals act as obstacles for the passage of gas; 3) presence of mesomorphic phases, whose local order can provide the best gas barrier properties [55, 56, 57]. Evidence of the presence of a mesomorph phase could be gathered via DSC analyses on 2,5‐2,4‐PHF (Table 2 and Figure 3), however its presence cannot be dismissed for 2,5‐2,4‐PTF and 2,5‐2,4‐PBF, since mesomorph phases are known to be challenging to identify by traditional means of analysis, due to their lower degree of order extending in short‐range domains, which in the case of furan‐based polymers are believed to be formed by pi‐pi interactions and hydrogen bonds between furan rings [21, 22].

**TABLE 4:** Gas barrier properties of 2,5‐2,4‐PTF, 2,5‐2,4‐PBF, and 2,5‐2,4‐PHF and their respective parent homopolymers [19, 47]. Data expressed as gas transmission rates with unit (cm^3^ cm)/ (m^2^ d atm). All measurements were carried out at 23°C. Data missing for 2,5‐PTF and 2,5‐PHF in humid conditions.

Gas	O_2_	CO_2_	O_2_	CO_2_
Relative humidity (%)	0	0	85	85
**PTF**				
**2,5‐PTF** [19]	0.022	0.029	—	—
**2,5‐2,4‐PTF**	0.138	0.063	0.274	0.113
**2,4‐PTF** [47]	0.138	0.122	0.078	0.073
**PBF**				
**2,5‐PBF** [19, 48]	0.104	0.080	0.091	0.095
**2,5‐2,4‐PBF**	0.018	0.018	0.099	0.0001
**2,4‐PBF** [47]	0.002	0.0007	0.003	0.002
**PHF**				
**2,5‐PHF** [19]	0.190	0.500	—	—
**2,5‐2,4‐PHF**	0.709	0.624	0.587	0.474
**2,4‐PHF** [47]	0.384	0.443	0.640	0.495

Observing the GTR values obtained with dry gases, it can be noted that the best performing materials were 2,5‐2,4‐PTF and 2,5‐2,4‐PBF, followed by 2,5‐2,4‐PHF. The results for 2,5‐2,4‐PTF and 2,5‐2,4‐PBF can be explained considering that they have a rigid and glassy amorphous phase, resulting in less free volume between the chains. Conversely, the results on 2,5‐2,4‐PHF were due to the combined effect of a mobile amorphous phase, characterized by high free volume, and a crystalline phase. The latter is typically responsible for high gas barrier, but it can also create high number of interphase boundaries, facilitating passage of gas in spite of the impermeability of individual crystallites [55, 56, 57]. The combination of interphase boundaries and lower free volume of the amorphous phase can explain why 2,5‐2,4‐PTF and 2,5‐2,4‐PBF displayed superior gas barrier than 2,5‐2,4‐PHF, in spite of being amorphous. Comparing 2,5‐2,4‐PBF with 2,5‐2,4‐PTF, it can be noted how the first was more performant, in spite of the two materials being apparently very similar in their microstructure, from what it was possible to observe with DSC and WAXS analyses (Table 2 and Figures 3 and 4). This result is consistent with what has already been observed in the literature for the reference homopolymers of 2,5‐2,4‐PAF, since the gas barrier properties in dry conditions of 2,5‐2,4‐PAF copolymers are intermediate between the ones found for the homopolymers of reference (Table 4 and Figure 6). 2,4‐PBF showed outstanding gas barrier properties in the literature, already explained by the potential formation of a more compact mesomorph phase [48]. If 2,4‐PBF repeating units could cause a similar effect in random copolymers, this would explain why the copolymer containing 2,4‐PBF units displayed greater improvements in gas barrier properties when compared to the copolymer containing 2,4‐PTF units.

Both CO_2_‐TR and O_2_‐TR were tested for the polymers under study, since oxygen and carbon dioxide interact with polyesters differently, and the overall permeability of each of the two molecules is the result of the balance between two effects, within the framework of the solution‐diffusion model of permeation [58]: 1) Size: oxygen molecules are smaller than carbon dioxide molecules, so their diffusion should be favored if steric hindrance had a predominant effect on diffusion, however, the path of smaller molecules through a polymeric matrix is also more tortuous, increasing the time needed to complete their diffusion; 2) Polarity: carbon dioxide molecules have no dipole moment, but they have two dipoles which can individually interact with polar ester moieties, and this can favor adsorption into polyesters, but also make desorption less favorable. The balance of both effects in polyesters typically favors the passage of carbon dioxide, making O_2_‐TR lower than CO_2_‐TR. However, this ratio was found to be inverted in furan‐based homopolymers in the literature (Table 4 and Figure 6), and is particularly conspicuous in the case of 2,4‐PAF homopolymers and 2,5‐2,4‐PAF copolymers. This can be attributed to a less favorable desorption of CO_2_ molecules from the matrix, or to a greater impact of steric hindrance on diffusion.

Lastly, for 2,5‐2,4‐copolymers, the gas barrier properties were tested in humid conditions: the presence of high relative humidity was found to have no significant negative impact. On the contrary, in the case of 2,5‐2,4‐PBF and 2,5‐2,4‐PHF, it even caused noticeable improvement. These results are particularly interesting, since water typically acts as a plasticizer, i.e., it increases the free volume, thus affecting the gas barrier properties of a polymer. However, in the case of furan‐based polyesters, the plasticizing effect can be mitigated, because water molecules may also form reversible interchain crosslinks based on hydrogen bonds, possibly enhancing the formation of a partially ordered mesomorph phase [19]. Depending on which of the two effects prevails (plasticization or reversible crosslinking), the gas barrier properties can be slightly improved or worsened, as shown in Table 4 and Figure 6. In one specific case, the effect of crosslinking caused a massive improvement in gas barrier properties: 2,5‐2,4‐PBF. This could be a signal that the intermolecular interactions in 2,5‐2,4‐PBF can be further optimized, as chains might be in a metastable state, which benefits from any change which can take them to a more interconnected, stable state. This corroborates what was observed during DSC scans (Figure 3), since 2,5‐2,4‐PBF showed a remarkably high physical aging phenomenon, which is typically correlated with the achievement of a state of greater conformational stability, once macromolecular rotations are unlocked at the glass transition temperature. In order to confirm this claim, DSC and WAXS analyses were carried out on 2,5‐2,4‐PBF 24 h before and 24 h after being subjected to gas barrier tests in humid conditions. The calorimetric and diffractometric curves were found to be unchanged. This proved that the crosslinking effect of water molecules prevailed over the plasticizing effect. In fact, if the latter prevailed, the T_g_ would have been lowered and a crystal phase might have developed, because of the widening of the temperature range in which crystallization was possible.

### 3.6 Sustainability Assessment and Potential Applications

Atom Efficiency Metrics were calculated setting the system boundary from furoic acid to 2,4‐PAF copolymers. When applicable, it included the following reactions:

1. Neutralization of furoic acid to potassium 2‐furoate;
2. Henkel‐type reaction on potassium 2‐furoate to FDCA isomers and furan;
3. Precipitation of FDCA isomers with HCl;
4. Hydrogenation of furan to HMF;
5. Conversion of HMF to 1,4‐BD;
6. Esterification of FDCA isomers;
7. Polymerization to 2,5‐2,4‐PAF.

The global reaction schemes to obtain 2,5‐2,4‐PAF copolymers with this route were found to be the following:

(2) $$\begin{array}{cc} & 2\;\;\mathrm{Furoic}\;\mathrm{Acid}+1,3-\mathrm{PD}+2\;\mathrm{KOH}+2\;\mathrm{HCl}\;\to \;2,5-2,4-\mathrm{PTF}\\ & \;\mathrm{Repeating}\;\mathrm{Unit}+\mathrm{Furan}+2\;\mathrm{KCl}+4\;H_{2}O \end{array}$$

(3) $$\begin{array}{cc} & 2\;\mathrm{Furoic}\;\mathrm{Acid}+2\;H_{2}+2\;\mathrm{KOH}+2\;\mathrm{HCl}\;\to \;2,5-2,4-\mathrm{PBF}\\ & \;\mathrm{Repeating}\;\mathrm{Unit}+2\;\mathrm{KCl}+3\;H_{2}O \end{array}$$

(4) $$\begin{array}{cc} & 2\;\mathrm{Furoic}\;\mathrm{Acid}+1,3-\mathrm{HD}+2\;\mathrm{KOH}+2\;\mathrm{HCl}\;\to \;2,5-2,4-\mathrm{PHF}\\ & \;\mathrm{Repeating}\;\mathrm{Unit}+\mathrm{Furan}+2\;\mathrm{KCl}+4\;\;H_{2}O \end{array}$$

Total Atom Economy, Organic Atom Economy, and Carbon Efficiency were calculated on these reactions using the following equations:

(5) $$\begin{array}{ccc} \text{Total Atom Economy} & & =(\text{Molar mass of desired product /}\\ & & \text{Total molar mass of reactants})\cdot 100 \end{array}$$

(6) $$\begin{array}{ccc} \text{Organic} & & \;\;\text{Atom Economy}=(\text{Molar mass of desired product}/\\ & & \text{Total molar mass of organic reactants})\cdot 100 \end{array}$$

(7) $$\begin{array}{ccc} \text{Carbon Efficiency} & & =\text{(Carbon atoms in desired product}/\\ & & \text{Carbon atoms in reactants)}\cdot 100 \end{array}$$

**FIGURE 7:**
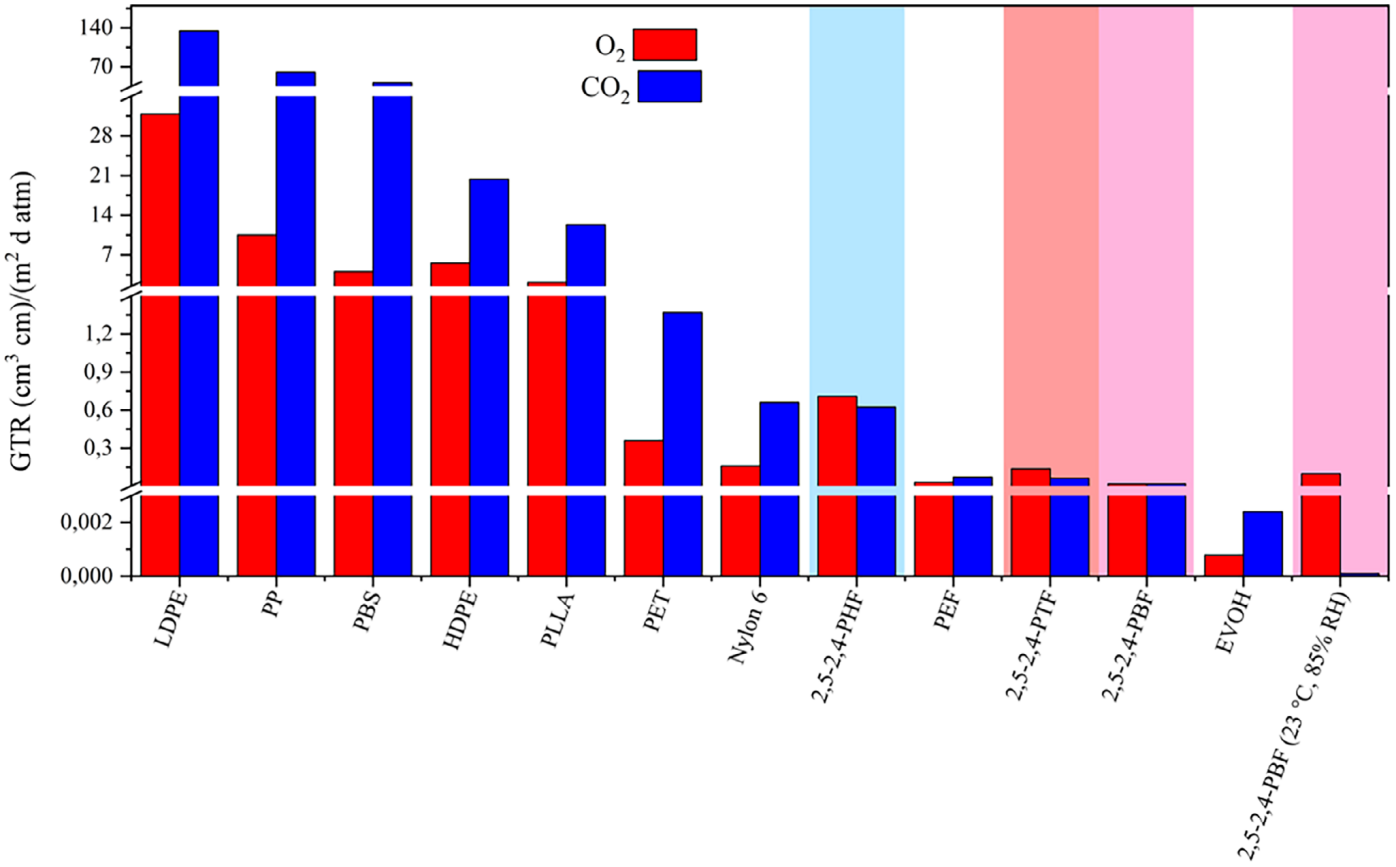
Gas Transmission Rates of 2,5‐2,4‐PTF (highlighted in red), 2,5‐2,4‐PBF (highlighted in pink) and 2,5‐2,4‐PHF (highlighted in blue), compared to commercial LDPE [59], HDPE [59], PP [59], PET [62], Nylon 6 [59], PLLA (98% L) [59], PBS [63], PEF [64], and EVOH (32% ethylene) [59]. All GTR values refer to dry testing conditions (23°C, 0% RH) unless otherwise specified.

The results, shown in Table 5, confirmed that the process for the production of 2,5‐2,4‐PBF had a perfect carbon efficiency, while the total atom economy could be increased with a straightforward improvement of salt management during a potential future process scale‐up. For example, the inorganic components could be readily integrated into circular recycling loops or upcycled as valuable by‐products (e.g., fertilizers). As a way to compare the potential performance of the materials under study for food packaging applications, the shelf life was evaluated as shown in the literature [59], and shown in Table 5. The results are a theoretical estimate which concretely shows the potential consequences of the outstanding gas barrier properties of 2,4‐PAF. For potential applications in semi‐rigid food packaging, the case considered was of a single‐material bottle 0.055 cm thick, with a surface area of 0.14 m^2^, containing 1 L of carbonated liquid with a CO_2_ concentration of 3500 ppm and an internal pressure of 2.75 atm, stored at 23°C. Given the CO_2_‐TR obtained in humid conditions (Table 4), a CO_2_ transmission higher than 20% determines the shelf life of the beverage [59], which was found to be exceptionally high for 2,5‐2,4‐PBF (Table 5), especially if compared with the shelf life of a biaxially oriented PET bottle, calculated in the same manner and using a CO_2_‐TR found in the literature [58] (Table 5). For potential applications in flexible packaging, the case considered was of a mono‐material film 0.005 cm thick, with a surface area of 0.10 m^2^, containing 200 g of fresh food products, such as meat, dairy, or vegetables, stored at 23°C. Given the O_2_‐TR obtained under humid conditions (Table 4), the transmission of O_2_ higher than 5 ppm determines the shelf life of the product [59], which was found to be exceptionally high for 2,5‐2,4‐PBF (Table 5), especially if compared with the O_2_‐TR found in the literature [58] for PVC kitchen wrap films, calculated in the same manner and using a O_2_‐TR found in the literature [58] (Table 5). The outstanding gas barrier performance of 25‐2,4‐PBF would guarantee exceptional shelf life for food products, dramatically decreasing food loss, food waste and the associated carbon emissions. Overall, the results of gas barrier tests highlighted the great potential of 2,5‐2,4‐PAF copolymers for the production of monomaterial food packaging. Unlike traditional multilayer polymer systems, which require energy‐intensive delamination or result in downcycled blends, the proposed monomaterial systems would simplify mechanical recycling by eliminating interfacial contamination. It can be hypothesized that the polymers under study would be recyclable, similarly to poly(ethylene furanoate) (PEF), which has been shown to be recyclable within preexisting infrastructure for poly(ethylene terephthalate) (PET), without negative impacts on recycled PET quality at the forecasted percentages of PEF market penetration [60]. Compared to other commercial polymers, 2,5‐2,4‐PAF copolymers had excellent gas barrier properties, as shown in Figure 7. It is particularly interesting to note that 2,5‐2,4‐PBF had improved CO_2_ barrier in humid conditions compared to poly(ethylene vinyl alcohol) (EVOH), which is a remarkable achievement, since EVOH is known to worsen its properties in contact with humidity and for this reason it has to be included in multilayer polymeric systems [59]. Thermally, 2,5‐2,4‐PAF had T_g_ values close to room temperature (about 43°C for 2,5‐2,4‐PTF, 38°C for 2,5‐2,4‐PBF and 10°C for 2,5‐2,4‐PHF), and this characteristic can be advantageous for flexible and semi‐rigid food packaging (such as films and containers), where high flexibility and toughness are prioritized. In comparison, PLA is known to have higher T_g_ (within the 50°C–60°C range) and it is notoriously brittle at room temperature [61], while 2,5‐2,4‐PAF copolymers exhibit remarkable toughness and elongation at break. The T_g_s of 2,5‐2,4‐PTF and 2,5‐2,4‐PBF are above room temperature, and this provides the necessary dimensional stability for packaging applications while avoiding brittleness. Just like PLA, these polymers would not be suitable for high‐temperature applications, such as contact with boiling water. However, this is not an issue for 2,5‐2,4‐PBF, which showed outstanding CO_2_ barrier performance even in humid conditions, making it an ideal candidate for carbonated beverage containers, which are typically stored at low temperatures. Regardless, in order to completely avoid any unwanted physical changes near room temperature, the materials under study could be compounded with the appropriate fillers or plasticizers to further modulate their T_g_.

**TABLE 5:** From top to bottom: Barrier Improvement Factor vs PET [62] calculated with GTR values obtained at 23°C, 0% RH; shelf life of flexible film for fresh food products and of rigid bottles for carbonated drinks estimated as shown in the literature [59].

	2,5‐2,4‐PTF	2,5‐2,4‐PBF	2,5‐2,4‐PHF
Sustainability metrics			
**Atom economy**	40	51	45
**Organic atom economy**	64	92	69
**Carbon efficiency**	69	100	75
**Barrier improvement factor**			
**O_2_‐BIF vs PET**	2.6	20	0.5
**CO_2_‐BIF vs PET**	22	76	2.2
**Theoretical shelf life**			
**Bottle for carbonated drinks (days)**	448	505874	107
**vs biaxially oriented PET (%)**	+157	+290632	−39
**Flexible film for fresh food (days)**	1.21	9.26	0.24
**vs PVC wrap film (%)**	+197	+2180	−42

## 4 Conclusions

This work presented a proof of concept for a sustainable integrated process of biorefining and polymerization starting from second‐generation biomass, to obtain potentially recyclable polyesters for the production of monomaterial food packaging. Specifically, the process was devised to minimize purification steps and the use of solvents, while maximizing its atom economy. Starting from 2,5‐DMF, 2,4‐DMF, 1,3‐PD, 1,4‐BD, and 1,6‐HD, three random copolyesters with high molecular weight were successfully synthesized: 2,5‐2,4‐PTF, 2,5‐2,4‐PBF, and 2,5‐2,4‐PHF. They were found to have tunable thermal, mechanical, and gas barrier properties depending on the glycol used, confirming the versatility of the process. DSC analyses found that by varying the glycolic subunit, it was possible to modulate the main thermal transitions, since 2,5‐2,4‐PTF and 2,5‐2,4‐PBF were found to be amorphous and glassy, while 2,5‐2,4‐PHF was found to be semicrystalline and rubbery. By means of DSC and WAXS analyses, an additional, partially ordered mesophase was hypothesized to be present inside the sample. In general, the molecular and thermal results highlighted excellent control over the structure of the copolymers and their high thermal stability. The mechanical properties of the copolymers under study were also highly influenced by the glycol used: 2,5‐2,4‐PTF was the most rigid and fragile, 2,5‐2,4‐PBF was rigid, but more ductile, and 2,5‐2,4‐PHF was the most flexible. The toughness of all copolymers was remarkably high. Finally, the gas barrier properties of the copolymers under study were found to be comparable to the ones of the homopolymers of reference, displaying excellent results both in dry and humid conditions. It should be noted that high relative humidity improved the gas barrier properties of 2,5‐2,4‐PBF toward CO_2_ of two orders of magnitude, making it nearly impermeable and suggesting applications in the field of packaging for carbonated beverages. Overall, the findings highlighted the great potential of these furan‐based copolyesters and of the process suggested for their production for the manufacturing of monomaterial, potentially recyclable, and sustainable food packaging applications.

## Author Contributions

E.B. conceptualization; polymer synthesis and characterization; data curation; data analysis; visualization; writing of the original draft. G.G. polymer characterization; correction and revision of the manuscript. M.S. conceptualization; supervision of the experimental activity; analysis of the overall experimental data; writing, correction, and revision of the manuscript. V.S. gas barrier measurements and data analysis; correction and revision of the manuscript. M.G. X‐ray diffraction measurements and data analysis; correction and revision of the manuscript. S.T. research funding; monomer synthesis and characterization; correction and revision of the manuscript. N.L. research funding; conceptualization; supervision of experimental activity; analysis of the overall experimental data; correction and revision of the manuscript.

## Conflicts of Interest

The authors declare no conflicts of interest.

## Supporting information




**Supporting File**: gch270102‐sup‐0001‐SuppMat.docx.

## Data Availability

The data that support the findings of this study are available from the corresponding author upon reasonable request.
