# Supplementary material for: Biobased Random Copolymers From Furandicarboxylic Acid Isomers: Proof of Concept for an Integrated Biorefinery Approach Toward Outstanding Sustainable Food Packaging
Source: Glob Chall. 2026 Apr 24;10(4):e00003. doi: 10.1002/gch2.202600003 (PMC13107169; doi:10.1002/gch2.202600003)
Supplement: Supplementary file 1 — Supporting File: gch270102‐sup‐0001‐SuppMat.docx. [file GCH2-10-e00003-s001.docx]

**Supplementary Information**

*for*

**Biobased random copolymers from furandicarboxylic acid isomers: proof of concept for an integrated biorefinery approach towards outstanding sustainable food packaging**

*Enrico Bianchi^a^, Giulia Guidotti ^a^, Michelina Soccio^a,b,c*^, Valentina Siracusa^d^, Shanmugam Thiyagarajan^e^ and Nadia Lotti^a,b,f^*

^a^Department of Civil, Chemical, Environmental and Materials Engineering, University of Bologna, Via Terracini 28, 40131 Bologna, Italy

^b^Interdepartmental Center for Industrial Research on Advanced Applications in Mechanical Engineering and Materials Technology, CIRI-MAM, Viale del Risorgimento 2, 40136, Bologna, Italy

^c^Interdepartmental Center for Industrial Research on Buildings and Construction CIRI-EC, Via del Lazzaretto 15/5, 40131, Bologna, Italy

^c^Department of Chemical Science, University of Catania, Viale A. Doria 6, Catania 95125, Italy

^e^Wageningen Food & Biobased Research, Wageningen University & Research, P.O. Box 17, 6700 AA Wageningen, the Netherlands.

^f^Interdepartmental Center for Industrial Agro-Food Research, CIRI-AGRO, Via Quinto Bucci 336, 47521, Cesena Italy

**corresponding Author*

4 Pages

4 Figures

**
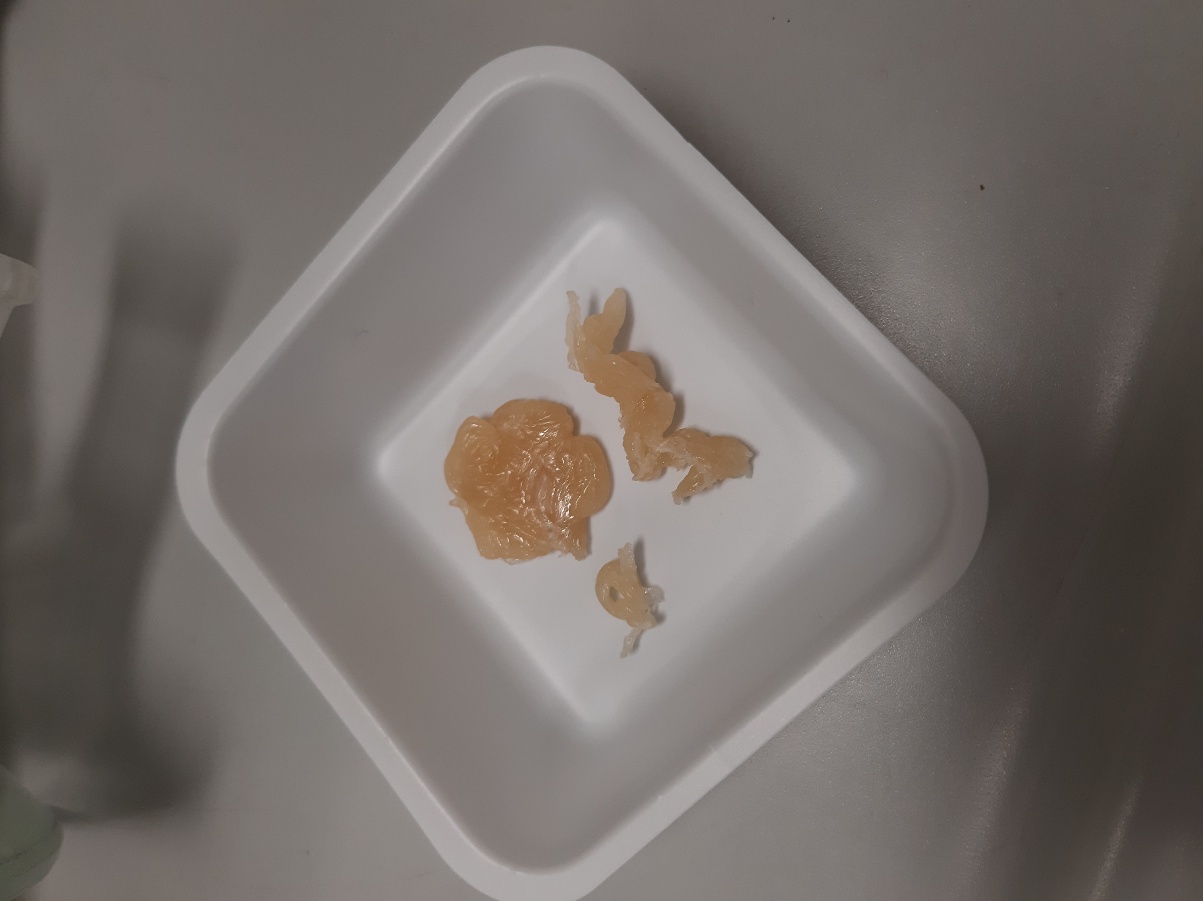
**

**Figure S1.** 2,5-2,4-PTF, as synthesized.

**
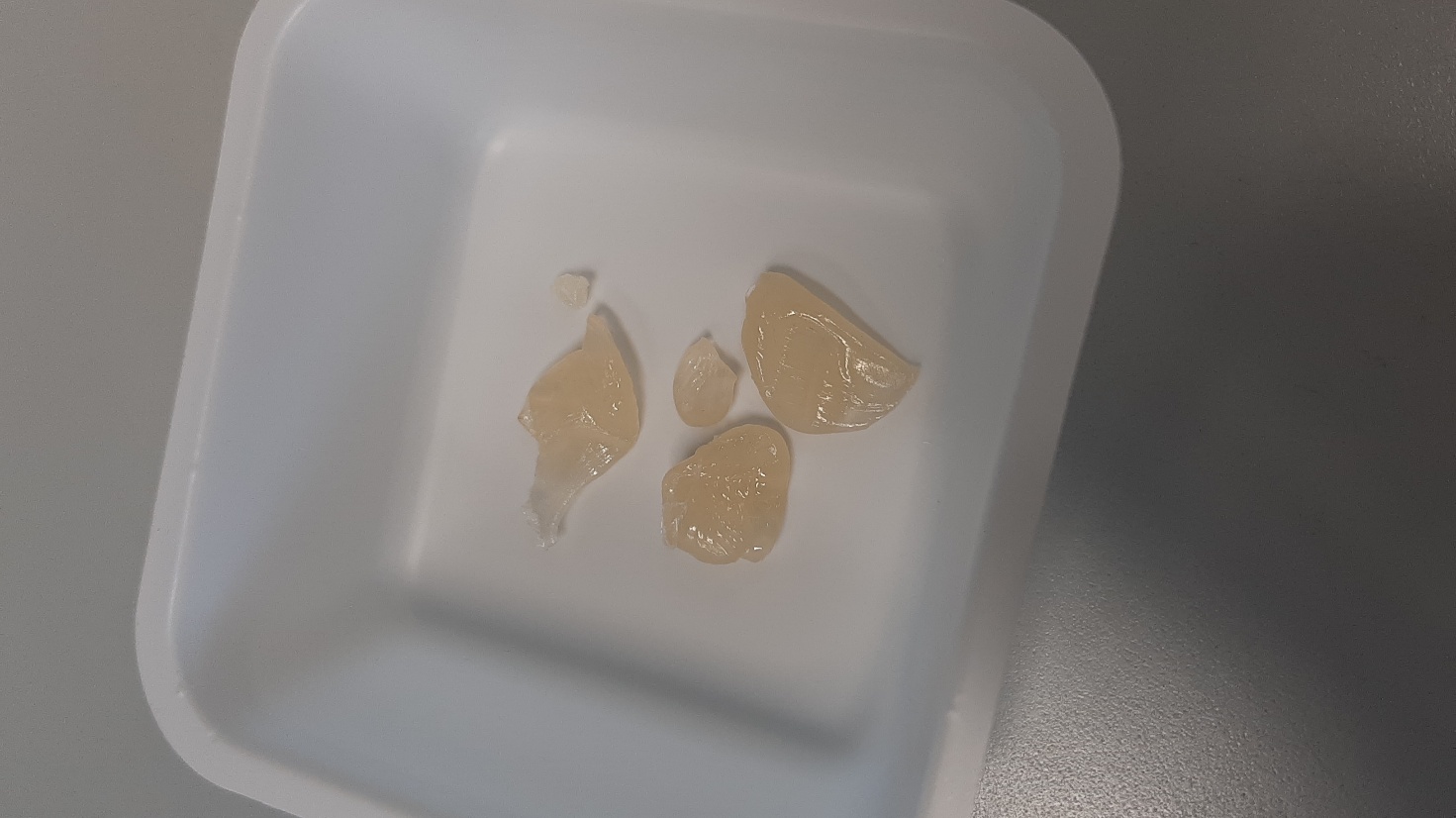
**

**Figure S2.** 2,5-2,4-PBF, as synthesized.

**
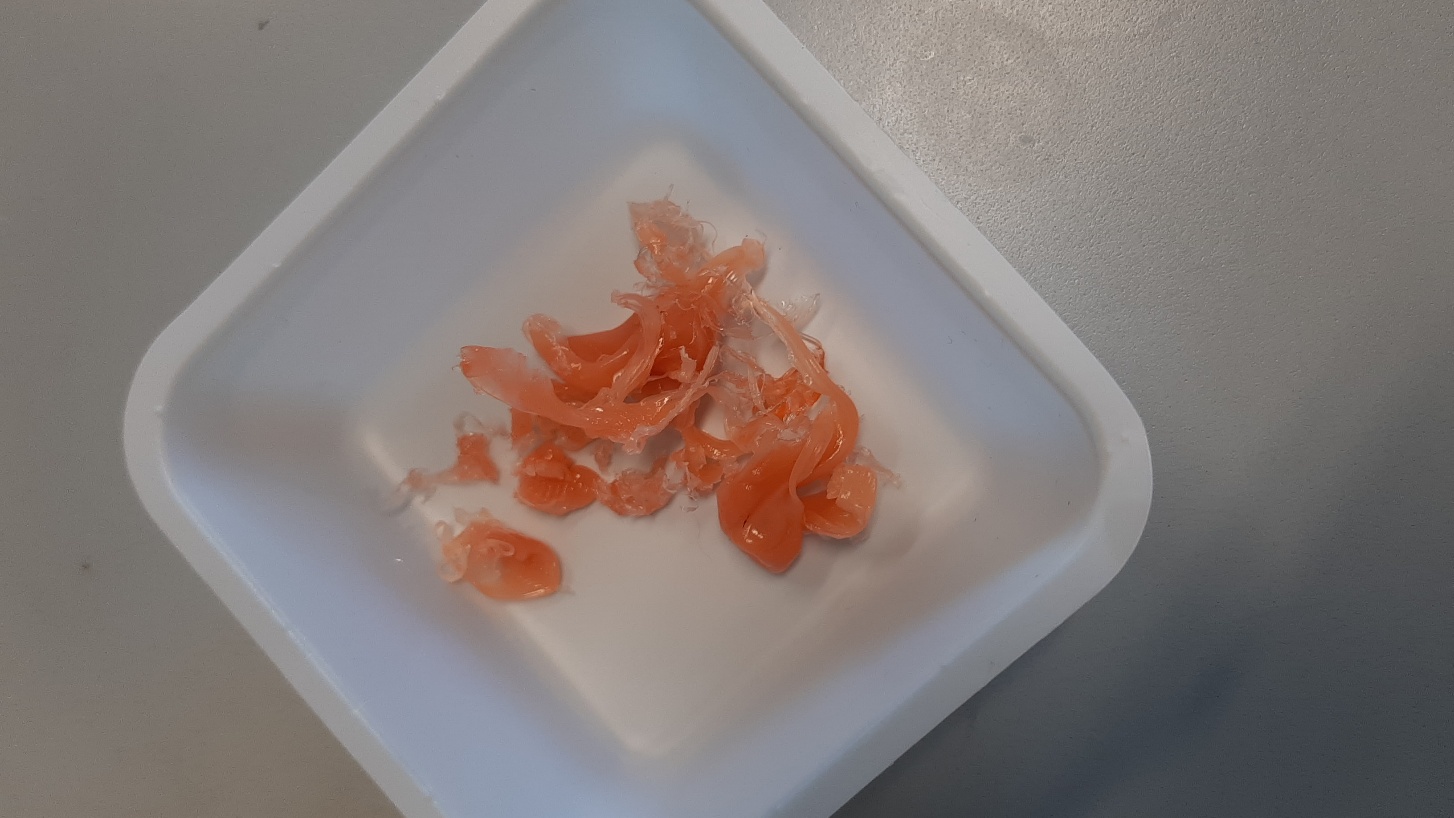
**

**Figure S3.** 2,5-2,4-PHF, as synthesized.


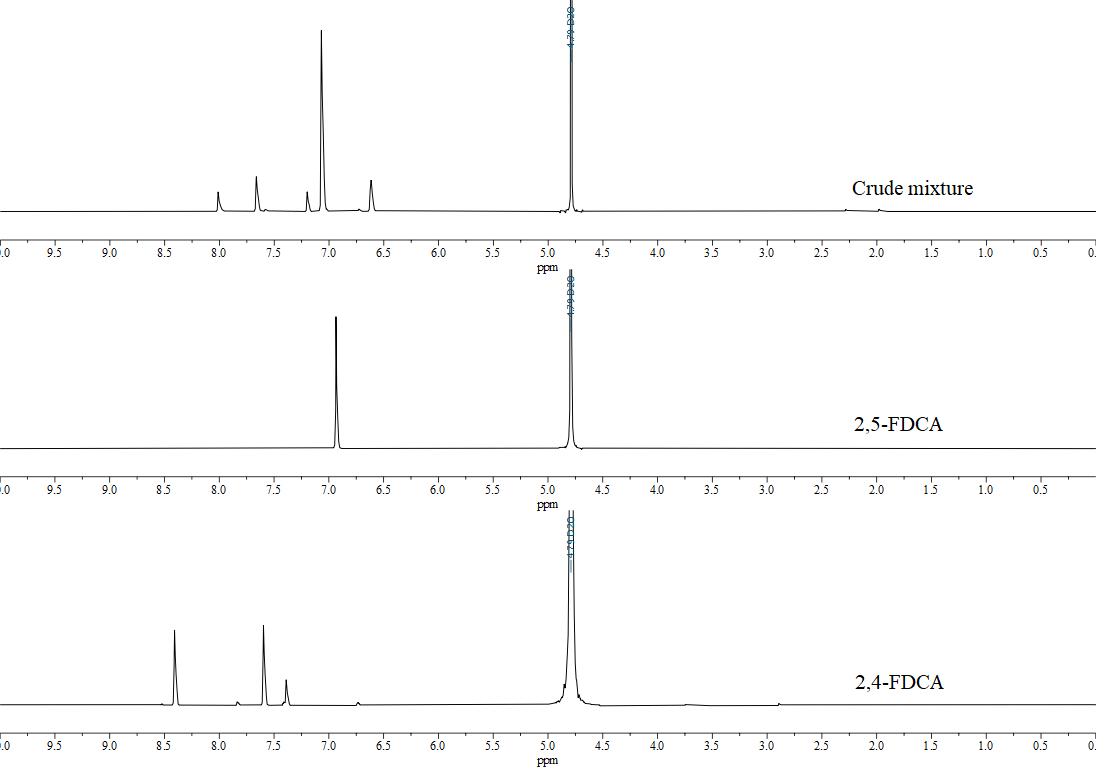


**Figure S4.** 1H-NMR spectra of the crude reaction mixture (containing 2,4-FDCA, 2,5-FDCA and unreacted potassium 2-furoate), of 2,5-FDCA and of 2,4-FDCA. All samples were dissolved in deuterated water.
